# Supplementary material for: Multidimensional health patterns and labor market participation among older workers: Evidence from a European six-year follow-up study
Source: PLoS One. 2025 Oct 8;20(10):e0333659. doi: 10.1371/journal.pone.0333659 (PMC12507312; doi:10.1371/journal.pone.0333659)
Supplement: S1 File — (PDF) [file pone.0333659.s001.pdf]

Supporting information

S1 File. Operationalization of the used health indicators

- Number of chronic diseases: Please look at card 6. Has a doctor ever told you that you had any of the conditions on this card? Please tell me the number or numbers of the conditions.
    - A heart attack including myocardial infarction or coronary thrombosis or any other heart problem including congestive heart failure
    - High blood pressure or hypertension
    - High blood cholesterol
    - A stroke or cerebral vascular disease
    - Diabetes or high blood sugar
    - Chronic lung disease such as chronic bronchitis or emphysema
    - Asthma
    - Arthritis, including osteoarthritis, or rheumatism
    - Osteoporosis
    - Cancer or malignant tumor, including leukemia or lymphoma, but excluding minor skin cancers
    - Stomach or duodenal ulcer, peptic ulcer
    - Parkinson disease
    - Cataracts
    - Hip fracture or femoral fracture
    - None
    - Other conditions, not yet mentioned
- Self-perceived health: Would you say your health is ...
    - Excellent
    - Very good
    - Good
    - Fair
    - Poor

For the analysis in the present paper we created a binary variable, combining: “Excellent” and “Very good” in a first category and “Good”, “Fair”, and “Poor” as a second category.

- Limitation in activities: For the past six months at least, to what extent have you been limited because of a health problem in activities people usually do?
    - Severely limited

    - Limited, but not severely
    - Not limited

- Depressive symptoms: In the last month, have you been sad or depressed?
    - Yes
    - No
