# Supplementary material for: Multidimensional health patterns and labor market participation among older workers: Evidence from a European six-year follow-up study
Source: PLoS One. 2025 Oct 8;20(10):e0333659. doi: 10.1371/journal.pone.0333659 (PMC12507312; doi:10.1371/journal.pone.0333659)
Supplement: S2 Table — (PDF) [file pone.0333659.s002.pdf]

Supporting information

S2 Table. Distribution of Latent Classes by Country (Row %)

**Country/MHP**	Overall healthy workers (1)	Workers with moderate limitations and low self-perceived health (2)	Physically healthy workers with low self-perceived health and depressive symptoms (3)	Workers in overall poor health (4)
Austria	5.5% (10)	52.2% (95)	30.2% (55)	12.1% (22)
Germany	6.0% (45)	44.3% (332)	34.7% (260)	15.1% (113)
Sweden	9.9% (36)	56.6% (205)	26.0% (94)	7.5% (27)
Spain	7.2% (20)	54.1% (151)	29.0% (81)	9.7% (27)
Italy	11.0% (28)	59.2% (151)	20.0% (51)	9.8% (25)
France	14.1% (60)	46.4% (197)	26.6% (113)	12.9% (55)
Denmark	8.7% (52)	67.4% (405)	18.1% (109)	5.8% (35)
Switzerland	9.5% (44)	63.6% (294)	21.0% (97)	5.8% (27)
Belgium	9.1% (34)	51.7% (194)	28.5% (107)	10.7% (40)
Israel	4.2% (5)	73.3% (88)	19.2% (23)	3.3% (4)
Czech	6.3% (25)	49.9% (199)	33.6% (134)	10.3% (41)
Republic	7.0% (10)	54.5% (78)	24.5% (35)	14.0% (20)
Luxembourg	5.8% (12)	49.8% (103)	36.7% (76)	7.7% (16)
Slovenia	8.3% (37)	39.2% (175)	35.0% (156)	17.5% (78)
