# Supplementary material for: Multidimensional health patterns and labor market participation among older workers: Evidence from a European six-year follow-up study
Source: PLoS One. 2025 Oct 8;20(10):e0333659. doi: 10.1371/journal.pone.0333659 (PMC12507312; doi:10.1371/journal.pone.0333659)
Supplement: Attachment — Submitted filename: RESPONSE_TO_REVIEWERS.docx [file pone.0333659.s004.docx]

**Response to Reviewers**

We thank the Editor and the two Reviewers for the valuable comments. Please find below our point-by-point responses.

**Reviewer 1**

| The authors address an interesting and socially relevant topic. They argue that health is a multidimensional construct and advocate for using a multidimensional health measure to examine the role of health in labor market participation at older working ages. Their approach represents a small but significant contribution to the field of health, employment and retirement research. The authors analyzed data from 5,006 older workers participating in the Survey of Health, Ageing and Retirement in Europe (SHARE), they performed a latent transition analysis based on four health indicators - chronic conditions, activity limitations, self-rated health, and depressive symptoms - to identify multidimensional health profiles (MHPs). In the second step, the examined the relationship between these MHPs and labor force participation six years later. Four distinct patterns were identified. The authors conclude that using health as a multidimensional construct allows to better understand the social implications of poor health. In addition to the research idea, I also appreciate the choice of dataset for this investigation. However, there are several major—primarily conceptual—issues with the paper that, in my view, must be addressed before it can be considered for publication. | We thank Reviewer 1 for their appreciation. |
| --- | --- |
| **Methdology**  The manuscript would gain from more clarity in the choice of the methodological approach and its description. | We thank the Reviewer for pointing out the need for greater clarity regarding the choice and description of our methodological approach. To enhance readability and transparency, we have revised **section 5.4 Statistical analyses**. We develop these efforts in detail further below (please see our response on the methodology of the latent transition analysis). |
| - Multidimensional construct: What is the author’s reasoning behind the indicators chosen for the mc? The fact that Boehme has chosen this? Why is this model superior to others? | In contrast to previously used models, Boehme’s model includes indicators of mental health. The inclusion of mental health indicators is highly relevant in the context of labor force participation in late careers. We made this rationale clearer by adding that the advantage of this model is that it includes indicators for mental health – unlike earlier models that focused uniquely on physical health – and that it combines health dimensions that have individually been shown to be relevant to labor force participation among older workers (see p. 4). |
| - Why using overlapping health concepts in the model? | The methodological approach we use to create the MHP allows to – and even welcomes – the inclusion of overlapping health concepts. This approach to allows examine how the different health concepts coincide. Unlike regression analysis, the methodological approach we use does not suffer from multicollinearity. We made this point clearer in section **5.3. Measures** on pp. 9-10. |
| - Why using dichotomous health indicators if linear indicators can also be used in latent group modelling? | We thank the reviewer for raising this important methodological point. We opted for dichotomous and categorical health indicators because our primary goal was to identify **qualitatively** distinct latent health patterns rather than quantitative gradations within a single dimension of health.  Dichotomization or categorization facilitates a clearer interpretation of the latent classes, making it easier to identify specific health profiles relevant to labor market participation. This approach aligns with previous latent-class and latent-transition analyses in aging research (e.g., Boehme et al., 2014; Ng et al., 2014) and supports our substantive interest in distinct subgroups characterized by combinations of health impairments rather than incremental differences along a continuum.  However, we agree with the reviewer that continuous indicators can offer additional granularity. Thus, we have added a brief reflection on this decision in Section **5.3 Measures** of the revised manuscript, explicitly acknowledging the trade-offs involved and suggesting continuous indicators as a valuable direction for future research (see p. 10). |
| - From my point of view the methodology latent transition analysis needs to be explained more in detail. How does one have to understand the clusters if the numbers of participants in each cluster differ by wave? How do you estimate belonging to a cluster? On which basis (wave 5 or 6? or a transition measure) did the authors calculate the distribution of the outcomes? | We thank the reviewer for pointing out the need for greater clarity regarding the choice and description of our methodological approach. To enhance readability and transparency, we have revised section **5.4 Statistical analyses** in the manuscript by explicitly stating the rationale behind selecting latent transition analysis (LTA). Specifically, we emphasized why LTA is particularly suitable for examining changes in multidimensional health patterns over time, as it allows capturing individual and structural stability across measurement points.  Additionally, we provided further clarification on the criteria used to determine the optimal number of latent classes (MHP), including details about statistical indicators (LL, BIC, sample-adjusted BIC, BLRT, and VLMR) and theoretical considerations.  The revised section now clearly articulates:   1. **The justification for choosing LTA**: emphasizing its appropriateness for longitudinal data and latent constructs (multidimensional health). 2. **The criteria and decision-making process**: detailing explicitly how we used both statistical fit indices and theoretical interpretability to identify the final latent classes. 3. **Clarification of statistical terminology**: ensuring all technical terms are clearly defined or explained in context to improve accessibility.   We believe these revisions significantly improve the clarity of our methodological approach. |
| - What does it mean for analyses and findings to use an initial sample with an age range from 50 to 60 years and following these for eight years, with the older reaching/passing statutory retirement age and the younger still being far away from it? | We thank the Reviewer for this comment. This age range may appear large, but is justified to achieve a large sample size. We conditioned the inclusion of participants in our sample on being employed/self-employed in the first wave of data we use. In addition, there exists a large amount of heterogeneity in timing of effective retirement across and also *within* European countries, which leads to strongly differing retirement trajectories (see e.g., (Madero-Cabib et al., 2021; Riedel et al., 2015).  For these reasons, we used a sample that is rather homogeneous in terms of labor force participation at baseline but heterogeneous eight years later.  We now mention this in the method section (see p. 8). |
| **Measurement of health** If concepts of health are in the focus of the paper, a detailed description, presentation and discussion of each health indicator chosen should be given. |  |
| - What is known about each measure? What are the underlying health concepts of each measure used? | The health measures we use have been extensively used in previous research. In the methods section 5.3. on Measures we now added a short paragraph to describe the health measures and to explain the relevance of each measure for labor force participation (see p. 10-11). |
| - What is known about stability over time, predictivity, validity? How were the measures operationalized – and why? Distributions in the sample.  - For example: is the term “depressive symptoms” justified if the underlying question is whether the respondent has “felt sad or depressed in the month before the interview”. Doesn’t the high proportion of participants shifting from Cluster 3 (wave 5) to Cluster 1 (wave 6) indicate that the question reflects a temporary condition, possibly linked to a specific event or living situation? | To address this comment, our manuscript now provides detailed information about the operationalization of the used health indicators in the Supplementary Material 1. For instance, we provide the full list of chronic conditions that have been assessed in the SHARE dataset. As mentioned earlier, if the variables assessed by SHARE were not categorical, we transformed into categorical variables. In addition, in Table 2, we now indicate the distribution of the measures in the overall sample.  The variable “depressive symptoms” indeed refers to the experience of the last month. It is therefore likely that it, for some study participants, reflects a temporary condition. We added a comment on this fact in the discussion section; we believe that fact underscores the usefulness of using a multidimensional health construct in examining the impact of health on labor market participation. |
| - The understanding of “self-perceived health” seems too limited in the manuscript. Why should it be an indicator for mental health? (p5) Several authors have reasoned about what this highly predictive indicator stands for. I recommend reading Jylhä 2009 “What is self-rated health and why does it predict mortality? Towards a unified conceptual model”. | We meant that self-perceived, or self-rated health also includes mental, as well as cognitive, aspects. But surely, we agree that it should be seen as a more global measure of health and we have now revised the manuscript accordingly in the presentation of each health measure, including the reference to the seminal paper by Jylhä. |
| - Number of chronic diseases: 1 vs 2+. Why this dichotomization? Which disease groups are included? | See above |
| **Further measures** - Measuring household income: Why not use "household net equivalized income," which more accurately reflects social status than total household income alone? This should be possible with SHARE data. | We thank the Reviewer for this comment. While we agree that "household net equivalized income" can provide a more nuanced picture of socioeconomic position, we in fact used **relative household income** categorized into quintiles within each country and not total household income as the reviewer indicated. This relative approach still provides a valid and meaningful indicator for identifying socioeconomically vulnerable households, as it effectively captures relative socioeconomic standing within national contexts and is commonly employed in comparative research using SHARE data (e.g., Mackenbach et al., 2008). |
| - Measuring change of weekly working hours. The finding is very surprising that on average, the workers have reduced their weekly working hours by >> 10 hours over 6 years. Which participants were included in the calculation: also those that had stopped working for full? In that case the overall mean is not useful for analysis. Or: was it due to some specific countries with special regulations allowing for such a substantial reduction of working time prior to retirement? This however, would raise the question of heterogeneity between countries. | In this analysis we included all workers, also those who had stopped working for full. The measure of working hours is used as an additional measure to job situation (with discrete answer options), contributing to a more nuanced understanding of the changes in labor force participation among older workers. We understand that the changes of working hours alone are not indicative, but together with the changes in job situation, they are. In fact, many older workers do not stop working for full but reduce their working hours to reduce the strains stemming from work. From our point of view the absolute number of hours is not very indicative, but the (in some cases significant) differences between the different MHPs in terms of working hours are. |
| **Cultural heterogeneity** - One important factor that may explain many of the findings in the manuscript is the strong country-level influence on the topics of (work,) health, employment, and retirement. These areas are highly context-dependent, shaped by national welfare systems, retirement regulations, social norms, and cultural perceptions of health. From my perspective, it is crucial for a study of this kind to discuss the potential implications of analyzing pooled data from 14 European countries as well as Israel. What does this heterogeneity mean for the findings? How are participants from different countries distributed across the identified clusters? Are there clusters predominantly composed of individuals from specific countries and vice versa? Were any sensitivity analyses conducted based on welfare state regimes or country groupings? | We thank the reviewer for raising this important issue which in the current manuscript is not sufficiently addressed. We agree that health, employment and retirement are indeed shaped by country-specific institutions and norms. In order to assess this aspect, we cross‑tabulated the four multidimensional health patterns (MHPs) with all 15 countries (N = 5 006). The χ² test indicated statistical dependence between MHP membership and country (χ² = 254.32, df = 39, p < 0.001); however, the effect size was small (Cramer’s V = 0.13), indicating that the relationship, though detectable, is weak in practical terms. Crucially, every country is represented in every MHP, and no single country accounts for more than 21.3 % of any class (Czech Republic in cluster 4). We note this as a limitation and suggest future multilevel or country‑specific mixture analyses.  The finding implies that class prevalence varies somewhat by country, but the latent structure itself is broadly comparable. We note this as a limitation and suggest future multilevel or country‑specific mixture analyses.  Revisions addressing this point have been inserted at four places in the manuscript:   1. Section 5.4, Statistical Analyses (Methods). A closing paragraph now explains the country‑by‑MHP cross‑tabulation procedure, reports the χ² and Cramer’s V statistics, and directs readers to the new supplementary table. 2. Section 6, Results. At the end of the paragraph describing the class characteristics, one sentence now reports the χ² (254.32, df = 39, p < 0.001), the small effect size (Cramer’s V = 0.13), and notes that no country exceeds a 21 % share in any class. 3. Section 7.1, Implications, Limitations, and Future Research (Discussion). The limitations paragraph has been expanded to interpret the small practical impact of the country effect, acknowledge the 21 % concentration, and propose multilevel or country‑specific mixture analyses as future work. 4. Supplementary Material. A new Table in the Supplementary Material 2 provides the full 4 × 15 country‑by‑MHP contingency table referenced in the main text. |
| **Overall** The authors acknowledge that their concept of "multidimensionality is still somewhat crude." I appreciate this critical reflection and would encourage the authors to reconsider their methodological approach. From my point of view not much is gained by specifically applying this approach, however, there are more differentiated ways of clustering health conditions in populations. Overall: did the approach applied really contribute to the central aim stated in the introduction: “These facts raise our principal question of what factors influence whether older workers with adverse health conditions exit or remain in the labor force.”? | As highlighted earlier, the method we use is appropriate for longitudinal data and latent constructs (multidimensional health). We emphasize our point of view in the manuscript by providing greater clarity regarding the choice and description of our methodological approach. |

**Reviewer 2**

| This manuscript addresses an important and timely topic: the identification of Multidimensional Health Profiles (MHP) among older workers and their association with employment status and working time reduction. The study uses high-quality longitudinal data from the SHARE survey and applies Latent Transition Analysis (LTA), which is an appropriate and advanced method to capture temporal health profile dynamics.  The findings offer valuable insights into the complex interplay between health and employment in later life, with clear implications for policy and active ageing strategies. The manuscript is clearly written and logically structured.  However, there are some areas that could be improved to enhance clarity and impact. | We thank the Reviewer for these comments. |
| --- | --- |
| -**Conceptual clarity** The definition of “Multidimensional Health Profile (MHP)” should be introduced more explicitly earlier in the introduction, before stating the hypotheses. | We have revised the introduction. A definition of MHP is now included in the introduction, on p. 4. |
| The formulation of hypotheses could be streamlined for better readability. | We thank the Reviewer for this comment. We now introduce all hypotheses using bullet points for better readability. |
| -**Methodology** Although LTA is well applied, the rationale for choosing this method over alternatives should be made clearer. | In section **5.4 Statistical analyses** we have now extended the manuscript by explicitly stating the rationale behind selecting latent transition analysis (LTA). Specifically, we emphasized why LTA is particularly suitable for examining changes in multidimensional health patterns over time, as it allows capturing individual and structural stability across measurement points. |
| The manuscript could benefit from a more critical discussion of the SHARE sample design, particularly regarding rotation and attrition across waves. | We addressed this point by stating in the limitation section that our finding of age differences in attrition rates with lower attrition rates among older workers may lead to an overestimation of individuals who are in retirement due to reaching statutory retirement age and thus and underestimation of the effect of poor health on labor force participation (p. 27). |
| -**Discussion and implications** The discussion could be strengthened by elaborating on the practical implications of the identified health profiles for policies on active ageing, employment support, and reducing inequalities. | We thank the reviewer for this comment. We complemented the discussion of the ALMPs with a practical example. The study we now mention, evaluates the efficacy of a small- group intervention promoting successful ageing at work among older workers in Germany. Such programs may contribute to improve mental health among older workers (p. 26). |
| It would also be valuable to comment on the potential cross-national variability and whether institutional differences may limit generalizability. | We appreciate this observation and have incorporated an explicit statement on cross‑national variability and its implications for generalizability. The manuscript now acknowledges that, although our pooled design yields an “average European” pattern, institutional differences could still influence the prevalence and labor market consequences of the identified multidimensional health patterns (MHPs).   - Section 5.4 – Statistical Analyses: A concluding sentence now explains that we assessed country‑level heterogeneity through a 4 × 15 cross‑tabulation of MHP membership by country, reporting χ² (254.32, df = 39, p < 0.001) and a small effect size (Cramer’s V = 0.13). - Section 6 – Results: We added a sentence noting that all countries contribute to every MHP and that the highest single‑country share in any class is 21 %. - Section 7.1 – Implications, Limitations, and Future Research: A new paragraph states that (i) the small Cramer’s V suggests limited cross‑national variability in class membership, yet (ii) institutional contexts may still moderate the strength of associations with labor‑market outcomes, and (iii) future work should employ multilevel or country‑specific latent‑class approaches to examine these institutional effects more directly.   These additions directly address the reviewer’s concern and clarify how potential cross‑national variability was evaluated and how it might affect the interpretation and generalizability of our findings. |
| -**Limitations** A more detailed reflection on sample attrition and its potential influence on MHP stability would be beneficial. | We addressed this point in the limitation section on p. 27 |
| The authors could acknowledge the reliance on self-reported health measures as a possible limitation. | We agree that self-reported (or as we call it (in line with the SHARE dataset) self-perceived) health, should be seen as a global measure of health (see e.g., Jylhä, 2009). However, the use of such as global measure was intended and we rather see it as a contribution to the vast literature on the measure than as a limitation. |
| This is a strong and relevant contribution to research on health and employment among older adults. The suggestions above are meant to improve clarity and robustness, but they do not challenge the study's overall validity. With minor revisions, the manuscript will be ready for publication. | We thank the Reviewer for their appreciation. |
